# A Floatable Piezo-Photocatalytic Platform Based on Semi-Embedded ZnO Nanowire Array for High-Performance Water Decontamination

**DOI:** 10.1007/s40820-019-0241-9

**Published:** 2019-01-24

**Authors:** Yaozhong Zhang, Xiaolu Huang, Junghoon Yeom

**Affiliations:** 1 Department of Electrical and Computer Engineering, Michigan State University, 428 S. Shaw Lane, East Lansing, MI 48824 USA; 2 Department of Mechanical Engineering, Michigan State University, 428 S. Shaw Lane, East Lansing, MI 48823 USA

**Keywords:** Semi-embedded structure, Photocatalysis, Piezocatalysis, ZnO nanowire arrays

## Abstract

**Highlights:**

- ZnO nanowires were securely immobilized onto a floatable photocatalytic platform, which had a uniform diameter (55 ± 5 nm) and length (1.5 ± 0.3 μm).
- An additional 20% of the probe pollutant (methylene blue) was degraded by piezocatalysis-assisted photocatalytic degradation.
- The crude oil pollutant was decomposed up to 20% within 6 h.

**Abstract:**

Photocatalytic degradation attracts considerable attention because it is a promising strategy to treat pollutants from industrial and agricultural wastes. In recent years, other than the development of efficient photocatalysts, much effort has been devoted to the design of reliable and inexpensive photocatalytic platforms that work in various environment conditions. Here, we describe a novel photocatalytic platform that is able to float and freely move atop water while performing photodegradation. Compared to common platforms, such as slurry reactors and immobilized photoreactors, the proposed platform is advantageous in terms of easy recycling and energy saving. Furthermore, the special configuration resulting from a two-step synthesis route, semi-embedded photocatalysts, addresses some of the remaining challenges, for instance, the contamination from the loose photocatalysts themselves. For the probe pollutant, methylene blue (MB), a reproducible and remarkable degradation activity of the platform, is observed and the effect of two primary factors, including surface area of the catalyst and mass transfer rate, is investigated. Besides, the piezo-photocatalysis effect, serving as an additional functionality, is confirmed to further improve the degradability of the platform, which offers an additional 20% of degraded MB. At last, the promising result of the degradation toward crude oil reveals the possibility of the platform to be used in gasoline pollution treatment.
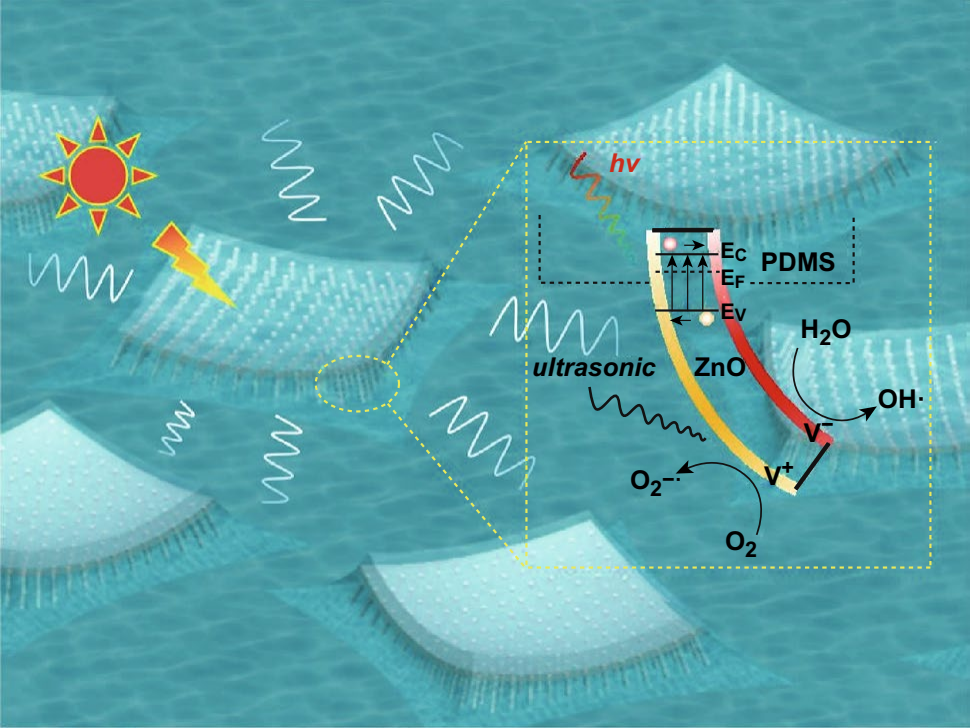

## Introduction

In the past few decades, water pollution has been becoming a serious global issue owing to the rapid development of the industry and widespread use of chemical and biological synthetic materials. Therefore, research on the aspect of pollutant removal for a natural water system is highly urgent and essential. Among numerous strategies, photocatalysis is one promising technology for disinfection and detoxification and has been demonstrated to be effective against various types of organic pollutants originating from industrial and agricultural wastes. This process mainly involves two reactions: oxidation and reduction. With the assistance of solar energy, photocatalysis is economical and applicable to versatile environmental conditions so that it has attracted considerable interest in recent years. For heterogeneous photocatalysis, the process is commonly realized by semiconductor photocatalysts, which can be categorized into two kinds in terms of their configurations: (1) slurry photocatalysts [1–3] and (2) immobilized photocatalysts. Among them, immobilized photocatalysts involve immobilized films/nanocomposites [4–6] and immobilized vertically grown nanowires (NWs) [7–9]. Slurry photocatalysts, including particles and 1D nanostructures, are the most commonly studied configurations, which are considered to possess high degradability owing to their large surface area-to-volume ratios and high homogeneity in aqueous solutions. With proper modification, excellent photocatalytic activities of these photocatalysts can be achieved, which are comparable to that of Degussa P25 [10–14]. However, some challenges such as easy agglomeration, complicated recovery from liquids, and increased absorption along the penetration depth of UV light hindered these photocatalysts from practical use. As an alternative route, immobilized film-like photocatalysts are preferable as practical options. This type of configuration possesses high recyclability, whereas their limited active surface area and mass transfer restraint become additional drawbacks. Recently, a photoreactor, especially microchannel-based photoreactor, has been suggested as one effective way to improve the performance of immobilized photocatalysts by increasing the surface-to-volume ratio and mass transfer and reducing the molecular diffusion distance. Such photoreactors serving as tools for the degradation of organic pollutants have demonstrated excellent reproducibility, stability, and degradability [15–18]. Moreover, a modification toward this design, such as employing immobilized NW photocatalysts, further improves its photodegradation rate owing to the larger active surface area in comparison with that of a film [19, 20]. However, most of these reactors require external power to drive the polluted solution to flow across the reaction chamber, and a specific platform is usually built to bring it closer to the light source, which inevitably increase the cost for scalable water cleanliness. In this regard, a configuration possessing immobilized NW-like photocatalysts both autonomously floating and freely moving atop the water is desired to improve the utilization of sunlight and overcome the mass transfer issue.

It is well known that for immobilized photocatalysts, a good support possesses features including reliable substrate-to-photocatalyst contact, stable property against generated oxidants, long lifetime in water, and environmental benignity. Furthermore, for a floatable immobilized platform, one more feature that needs to be taken into account in fabrication is a lightweight. Polymers were found suitable to use as floatable substrates [21–23]. Therefore, in this study, this novel configuration utilized a polymer as a substrate. Owing to the advantages of cost-effectiveness, substrate compatibility, and excellent degradability, ZnO was employed as the photocatalyst [24–27]. To our knowledge, a floatable photocatalyst is rarely explored. This work proved the concept of a photocatalytic platform that was able to address the aforementioned problems that other platforms encountered.

In detail, a two-step synthesis route (hydrothermal growth on a rigid substrate and a second hydrothermal growth on a flexible substrate) enabled a ZnO NW array to be securely and uniformly fixed onto the polymer substrate (ZP film). As a result, the light and flexible polymer allowed the ZP film to freely float and move on top of water, while the semi-embedded configuration tackled some problems such as re-suspension and light utilization. Besides, the investigation of the mechanical property and thermal stability confirmed the high durability and reusability of the ZP film to harsh environments. In the photodegradation experiment, the surface area and mass transfer, as two primary aspects, were found affecting the photodegradation of the ZP film. The enhanced photocatalytic activity was obtained at a large NW length and high mass flow rate. Moreover, the piezo-photocatalytic effect, as an additional functionality, was demonstrated to further improve the degradability of the ZP film by around 20%. Other than methylene blue (MB) dye, the pollutant removal capability of the ZP film toward crude oil was confirmed, which revealed the potential of the ZP film in use for various industrial wastes in open water systems.

## Experimental

### Initial Growth of ZnO Nanowire Array

All the chemicals are of analytical grade and used without further purification. A ZnO NW array was synthesized via the hydrothermal method as previously described [28]. In the first step, 10 mM Zn(CH_3_COO)_2_·2H_2_O (99.9% Sigma-Aldrich) was dissolved in methanol (Fisher Scientific). After vigorous stirring at 60 °C for 2 h, the mixed solution was spin-coated onto a glass substrate at 1000 rpm for 30 s. Prior to the seed deposition, the glass substrate was cleaned by acetone, isopropanol alcohol, and deionized (DI) water, consecutively. The seeded glass substrate was heated to 150 °C for 1 h for dehydration. The growth solution of 25 mM Zn(NO_3_)_2_·6H_2_O (Sigma-Aldrich) and 25 mM hexamethylenetetramine (HMTA, Sigma-Aldrich) was prepared and preheated in a convection oven at 90 °C for 1 h to achieve thermal equilibrium. The annealed substrate was then immersed into the growth solution at 90 °C for 6 h. Consequently, a vertically grown ZnO NW array was obtained on the glass substrate after thoroughly rinsing by DI water.

### Secondary Growth of ZnO Nanowire Array on PDMS

In the second step, a certain amount of polydimethylsiloxane (PDMS) solution (10 parts of SYLGARD 184 pre-polymer and 1 part of the curing agent, Dow Corning Co.) was fully covered and spin-coated on the NW-grown glass substrate. The spin coating speed was varied in terms of the desired thickness of the PDMS layer. For example, spin coating at 1000 rpm for 90 s produced a PDMS film with around 300 µm thickness. To avoid potential defects, the spin coating speed for the PDMS layer should not exceed 3000 rpm. The PDMS layer was thermally cured at 60 °C for 12 h and cooled to the room temperature overnight. The PDMS layer was then detached from the glass substrate. To facilitate the peeling of PDMS (extremely thin to be handled on its own), a polyethylene terephthalate (PET) film was utilized as a backing layer. The PET film was brought into intimate contact with the PDMS layer prior to its detachment. The as-fabricated PET/PDMS stack was placed atop the ZnO growth solution with the embedded NW array facing down at 70 °C for 12 h. The sample was thoroughly rinsed with DI water and completely dried. Finally, the PET film was separated from the PDMS layer to obtain a vertically aligned ZnO NW array on the PDMS membrane (denoted as the ZP film).

### Material Characterization

The morphology, crystallinity, and composition of the ZnO NW array both on the glass and PDMS substrates were characterized by scanning electron microscopy (SEM, JEOL 6610LV, Hitachi S-4700II), transmission electron microscopy (TEM, TALOS F200X), X-ray diffraction (XRD, Bruker-AXS), energy-dispersive X-ray spectrometer (EDS, JEOL 6610LV), and photoluminance spectrometer (PL, Ocean Optics, Inc. USB spectrometer and optical fiber) with the excitation laser at 337 nm.

### Photocatalytic/Piezocatalytic Assessment and Activities

#### Immobilized, Floating Photocatalytic Platform

Photocatalytic degradation was carried out in an aluminum foil-sealed case with a magnetic stirrer stage inside. Two 9 W florescent tubes (Philips *λ* = 375 nm) were placed 30 cm above the dye solution container. A 10 mM methylene blue (MB, 1.5%, Sigma-Aldrich) aqueous solution was prepared as a model polluted water. Four pieces of the ZP film (1 × 1 in.^2^ each piece) were utilized afloat on top of 40 mL of the dye solution (with the ZnO NW side facing down). Prior to irradiation, the dye solution was kept in dark for 30 min to establish the absorption/desorption equilibrium. The UV light irradiation was established perpendicular and with a distance of 30 cm to the floating ZP film. One milliliter of solution was extracted from the container every 30 min as the photodegradation progressed. The extracted solution was centrifuged at 10,000 rpm to remove any impurities and examined for absorbance in a UV–Vis spectrometer (Lambda). The absorption peak at 667 nm corresponded to MB, and the peak height can be correlated with the MB concentration. The photodegradation experiment was repeated three more times under the same working condition to test reusability/recyclability. The same degradation experiment (but only one 1 × 1 in.^2^ ZP film used for comparison) was carried out in 20 mL MB solution to evaluate the role of the secondary growth of the ZnO NW array on its photocatalytic performance. The effect of the mass transfer on the floating immobilized ZP film was investigated by turning on or off the magnetic stirrer during the degradation experiments.

#### Mechanical Stability of ZnO NW Array on PDMS: Piezocatalytic Degradation

The mechanical stability of the two-step grown ZnO NW array on the PDMS film was tested using the standard tape testing and compared to a ZnO NW array directly grown on a PDMS film. The bendability and stretchability of the PDMS film allowed the ZP film to be placed on a curved surface with a small radius of curvature (i.e., an 8-mm-diameter glass rod). The rod mounted with the ZP film was connected to the overhead mixer (Dragon Lab, OS20-S) and tested for photodegradation. The piezocatalytic effect of the ZP film was tested in an ultrasonic bath (Fisher Scientific, 160 W, 40 kHz), as a floating catalytic configuration and/or an overhead mixer configuration. One piece of the ZP film (1 × 1 in.^2^) was utilized for piezocatalytic degradation experiments to treat 40 mL of the dye solution. Photocatalysis-assisted piezocatalytic degradation was also performed in the presence of UV irradiation (the same condition as stated above).

#### Crude Oil Degradation

The floating ZP film was tested for crude oil degradation. Each sample had 50 mg of crude oil (Saudi Arabian Berri crude oil) dispersed on 2 mL DI water. One piece of the ZP film (circular, 16 mm diameter) was placed on top of the oil solution. After degradation, 5 mL of petroleum ether (Sigma-Aldrich) was used to dilute the remaining crude oil prior to the UV–Vis spectrometry measurement.

## Results and Discussion

### Two-Step Synthesis of ZnO NW Array on PDMS

In a typical hydrothermal synthesis, the substrate is deposited with a seed layer by various means prior to the growth of vertically aligned ZnO NW array. The structural stability of the NW array on the substrate is, thus, highly dependent on the adhesion between the seed layer and substrate. When a rigid inorganic substrate (e.g., glass, silicon) is used, the seed layer can be thermally treated at relatively high temperatures (often over 200 °C), which significantly improves the adhesion. However, this high-temperature treatment cannot be applied for polymeric substrates. Moreover, polymeric substrates are flexible and bendable, making it easy for the seed layer to be delaminated. Therefore, the NW array directly grown on a polymer substrate typically suffers from weak adhesion and is easy to be detached from the substrate. Our approach to improving the NWs’ adhesion to the polymer substrate is to utilize a two-step hydrothermal growth and secure the NW array by partially embedding the NWs in the polymer matrix.

**Fig. 1:**
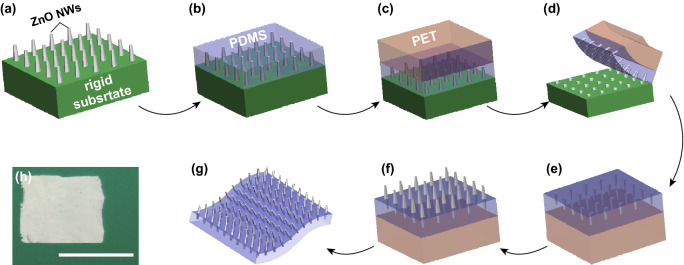
Schematic of the proposed two-step fabrication process of the vertically aligned ZnO NW array on a polymer substrate. **a** A ZnO NW array was vertically grown on the glass slide via the hydrothermal synthesis. **b** A certain amount of a PDMS solution was spin-casted onto the ZnO NW array and thermally cured afterward. **c** A PET film was brought into intimate contact with the PDMS film. **d** The PDMS/PET stack was detached from the glass slide simultaneously. **e** The PDMS/PET layer became flat owing to the recovery of the PET film. **f** The ZnO NW array embedded in the PDMS film was regrown to a desired length via the same hydrothermal process. **g** The ZP film obtained after the PET film was removed from the PDMS film. **h** Photograph of the as-prepared ZP film (1 × 1 in.^2^). Scale bar: 1 in

Figure 1 shows a schematic of the proposed two-step growth method to fabricate a robust ZP film, i.e., vertically aligned ZnO NW array strongly attached to the polymer (PDMS) substrate. In the first synthesis step (Fig. 1a), a vertically aligned ZnO NW array with the desired aspect ratio (*W*/*L* = 0.04–0.08, 60 nm in diameter, 2 μm in length) is grown from a seeding layer on a glass substrate [29]. A thin PDMS layer of relatively uniform thickness is then spin-casted onto the ZnO NW array-covered surface to fully encapsulate the NW array (Fig. 1b). Here, the thickness of the PDMS layer can vary from 200 to 500 µm depending on the different spin coating speeds. Prior to the detachment of the PDMS layer from the glass substrate, a PET film is brought into conformal contact with the PDMS surface to increase the stiffness of the PDMS layer (Fig. 1c, d). The PET film is employed because the thin PDMS layer is susceptible to damages during detachment from the glass substrate. While the molded PDMS film encapsulates the majority parts of the ZnO NW array, the NW tips remain exposed and serve as a seeding layer for regrowth. The PDMS/PET stack is placed on top of the same growth solution with the NW side facing down for the secondary hydrothermal synthesis (Fig. 1e, f). Because PDMS/PET is lighter than water, the stacked film is afloat during the second growth step. The standalone ZP film is obtained after the separation of the PET film (Fig. 1g). A photograph of the as-grown ZP film with the proposed two-step process is shown in Fig. 1h. In the single-step hydrothermal synthesis, the adhesion strength of the NW array to the substrate is determined by the interfacial strength between the seed layer and substrate. Therefore, longer NWs are difficult to be grown because they are easily detached from the substrate. In our proposed two-step synthesis approach, parts of the NWs are securely anchored onto PDMS, allowing the NWs to grow much longer than the ones generated by the single-step process.

**Fig. 2:**
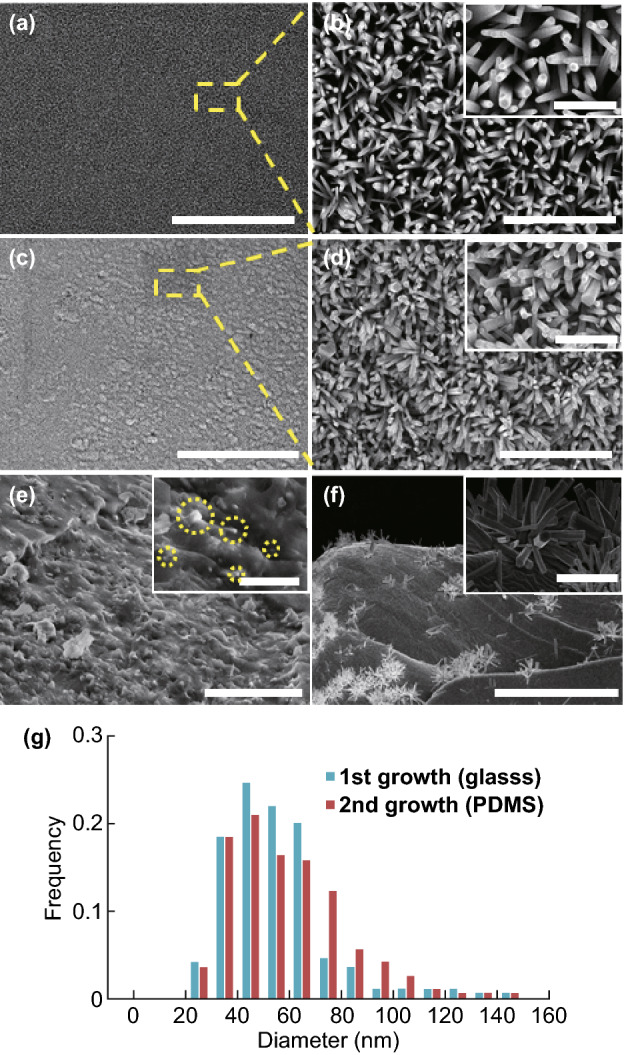
SEM images of the ZnO NW array (**a**–**b**) on the glass substrate after the first growth, (**c**–**d**) on PDMS after the secondary growth, **e** on the PDMS substrate (~ 300 μm in thickness) before the secondary growth, and **f** directly grown on the PDMS substrate. **g** Size (diameter) distribution of the ZnO NW array after the first and second growth. Scale bar: **a**–**d** 50 μm in the large view and 3 μm in the inset, **e** 20 μm in the large view and 5 μm in the inset and **f** 50 μm in the large view and 5 μm in the inset

**Fig. 3:**
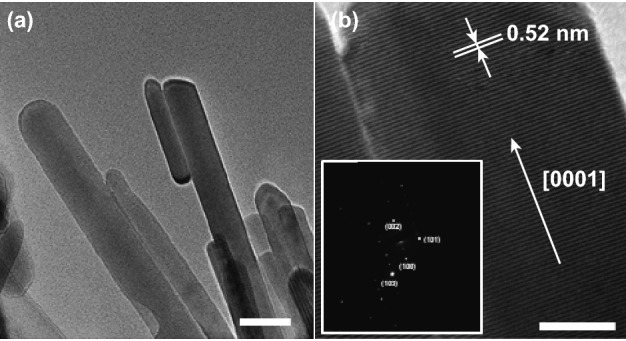
**a**–**b** TEM images of the ZnO NWs on a ZP film. The insert in **b** is electron diffraction (SEAD) pattern. Scale bar: **a** 50 nm, **b** 10 nm

Figure 2 shows the SEM images of the ZnO NW array at each key process. In Fig. 2a, b, it can be observed that the highly ordered ZnO NW array is uniformly distributed over the glass substrate after the initial growth. The average diameter and length of the ZnO NWs are estimated to be 60 ± 5 nm and 2 ± 0.3 μm, respectively. The values in parenthesis are the standard deviation from the size distribution. Figure 2e shows a detached PDMS film with the encapsulated ZnO NW array before the secondary growth, showing that the tips of the broken ZnO NWs are exposed (see the yellow circled regions in the inset image) and can serve as a fresh seeding layer. Moreover, the regrown NW array on the PDMS substrate (Fig. 2c, d) preserves the NW density, which is 121–132/9 μm^2^ in comparison with 100–110/9 μm^2^ on the glass substrate (see Fig. 2a), demonstrating the successful regrowth of the majority of the original ZnO NW array. The average diameter and length of the ZnO NWs after the secondary growth are 55 ± 5 nm and 1.5 ± 0.3 μm, respectively. The size (diameter) distributions of the ZnO NWs after the first and second growth are shown in Fig. 2g. The average NW diameter becomes slightly larger after the secondary growth. The hexagonal shape of the ZnO NWs is retained during the first and second growth steps, following the same crystalline orientations. A ZP film in a failed detachment is shown in Fig. 2f, where a sparse NW array is obtained. The morphology of the as-prepared ZnO NWs on both the glass substrate and PDMS layer is highly affected by factors such as reaction duration, temperature, and solvent concentration as reported in other literatures [30–32]. In contrast, the ZnO NW array directly grown on the PDMS substrate via the single-step synthesis exhibits a low density, indicating that the NWs may have been detached from the surface during the post-processing (such as rinsing and handling) owing to their poor adhesion (see Fig. 2f). To further investigate the morphology and crystal structure of the regrown ZnO NWs on the ZP film, they were scratched off and dispersed in ethanol for TEM imaging. As displayed in Fig. 3a, the diameters of the ZnO NWs are observed around 40 nm. Notably, the regrown ZnO NWs have a uniform diameter along the growth direction. The clear fringes with spacing at 0.52 nm and sharp spots in the electron diffraction (SEAD) pattern in Fig. 3b are found matching with those of the ZnO wurtzite structure [33], for which [0001] is the preferred growth direction.

### Material Characterization

**Fig. 4:**
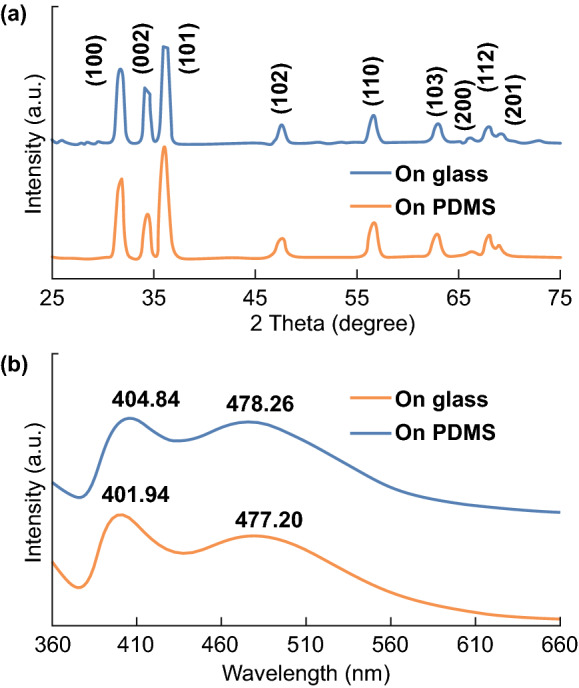
**a** XRD patterns of the ZnO NW array. **b** Photoluminescence spectrum of the ZnO NW array

The crystallinity of the ZnO NWs on the glass (after the first growth) and PDMS (after the secondary growth) substrates was characterized by XRD analysis and photoluminescence (PL) spectroscopy. As depicted in Fig. 4a, the XRD pattern of the ZnO NW array is recorded from 25° to 75°. It is clearly seen that each diffraction peak from the glass substrate has a corresponding peak from the PDMS substrate at the same position, implying that the proposed two-step process offers a coherent crystalline structure of the ZnO NWs. Additionally, all the diffraction peaks on both the substrates can be indexed to the wurtzite ZnO phase, where the peaks with a strong intensity indicate a c-axis-preferred orientation of the ZnO NWs [8, 12]. The PL spectrum of the ZnO NW array on both the substrates (see Fig. 4b) has a good agreement with the previous reports [7, 11], where the near-band-edge (NBE) emission and defect-related peaks are observed around 400 and 490 nm, respectively. The intensity of the defect-related peak is comparable to that of the NBE peak, indicating the significant defect level of the NWs. The defective NWs may be beneficial for their photocatalytic activity owing to the decreased surface state-related recombination of the photogenerated electron–hole pairs [11]. Interestingly, the ratio of the NBE peak to the defect-related peak on glass is close to that on PDMS, which also reveals the coherent crystallinity of the ZnO NWs after the secondary growth. Both the XRD analysis and PL spectrum confirm that the two-step process serving as an in situ growth method enables the ZnO NWs to grow consistently with a predetermined state.

### Structural Integrity of ZnO NW Array on PDMS

**Fig. 5:**
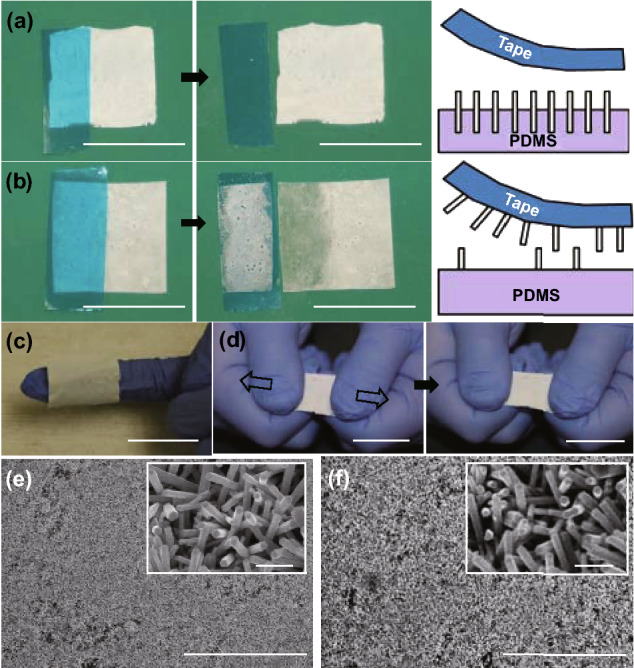
**a**, **b** Photographs and schematics of the tape test on a ZP film (1 × 1 in.^2^) and direct ZnO NW array growth film (1 × 1 in.^2^). **c**, **d** Photographs of a ZP film wrapped on a finger and stretched to more than 50% of the original length, respectively. **e**, **f** SEM images of a ZP film after tape test and other chemical and mechanical tests. Scale bar: **c**–**e** 1 in, **e**–**f** 50 μm in the large view and 1 μm in the inset

The long-term performance of the immobilized photocatalytic system is governed by the stability and adhesion of the photocatalysts to the immobilizing substrate. In particular, a floatable immobilized photocatalytic platform, which is of interest in the present study, entails additional requirements for the immobilizing substrate, including transparency to near UV and visible light and a density lower than that of water. The latter constraint necessitates the usage of polymeric or organic substrates. The previous fabrication approaches for incorporating photocatalysts onto polymer substrates for floating immobilized photocatalytic systems included physical vapor deposition [34], electrospinning [35], and spray/dip coating [36]. However, the photocatalysts are likely to fall off from the substrate during handling and repeated operation owing to the weak adhesion. To further prove the excellent structural stability of the proposed immobilized system, we subjected the ZP film to various operating conditions such as bending, stretching, contact, and temperature variation. Figure 5a shows the tape test conducted on a 1 × 1 in.^2^ ZP film. A strip of a clean dicing tape is placed on top of the ZP film, where the ZnO NW array is contacted intimately to its adhesive side. After peeling away from the ZP film, we inspect the tape and find only a few white dots on its adhesive surface. The microscope inspection revealed that majority of the ZnO NWs remained on the substrate after multiple tape tests, confirming the expected stability of the ZP film. In comparison, the tape tests were conducted on another immobilized ZnO film in which the NW array was directly grown on the PDMS substrate. As shown in Fig. 5b, a large number of ZnO NWs are pulled off by the tape, which can be attributed to the weak adhesion between the NWs and substrate. Refer to the schematics in Fig. 5a, b, which illustrate that the partially embedded ZnO NW array created by the two-step growth method provides a strong adhesion to the PDMS substrate, whereas the surface-grown NW arrays can be easily damaged or disturbed by external contacts.

This unique configuration of the ZnO NW array on PDMS allows the immobilized film to be bent and stretched without undermining the structural integrity. Figure 5c, d shows a ZP film wrapped on a finger and stretched to more than 50% of the original length, respectively. If the NW array is deposited or directly grown onto the PDMS surface, such bending or stretching of the substrate may cause the delamination of the NW array. In the ZP film, the individual ZnO NWs are vertically oriented and strongly anchored onto PDMS; therefore, the NWs appear to be intact in spite of such harsh handling (see Fig. 5e). Finally, no apparent morphological change is observed (see Fig. 5f) in the ZP films that are immersed in the common solvents (e.g., water and alcohols) and subject to vigorous mixing conditions or various temperature fluctuations (60 °C for 12 h). We believe that this superior structural robustness of the ZnO NW array on PDMS will present a reliable immobilization platform and well accommodate the harsh environments for practical applications.

### Photodegradation: Floatable Immobilized Photocatalytic Platform

**Fig. 6:**
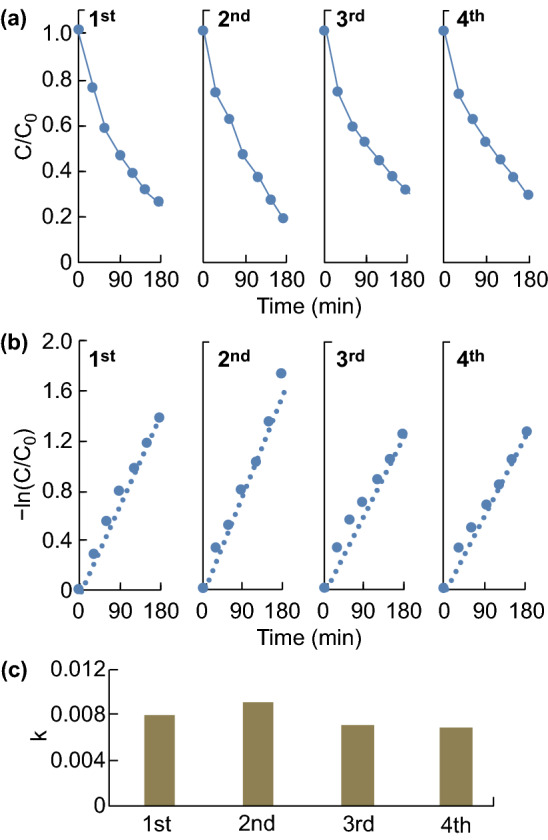
**a** Degradation profiles of the MB dye (at 667 nm) in four consecutive cycles. **b** Photodegradation kinetics of the MB dye in four consecutive cycles. **c** Constant *k*

ZnO nanostructures have been extensively studied as photocatalysts in the last few decades [2, 24]. Upon receiving photons with energy higher than the band gap of ZnO, the ZnO NWs fixed on the PDMS substrate are capable of generating electron–hole pairs, which convert O_2_ and H_2_O into O_2_^−^· and ·OH, respectively. These oxidants are able to decompose organic pollutants into smaller molecules and eventually into water and carbon dioxide [2, 3, 24]. Here to investigate the photocatalytic activity of the ZnO NW array on a PDMS film, MB was chosen as a probe pollutant, and its time-dependent decomposition under UV irradiation (~ 375 nm) was measured using a UV–Vis spectrometer. In this experiment, the ZP films were placed afloat with the ZnO NW array facing down toward the MB solution. Because PDMS is transparent to near UV [37–39], the UV light can penetrate the PDMS layer and excite the electron–hole pairs underneath the ZnO NW array. After a 3-h-long experiment, the ZP films were dehydrated and reused for photodegradation in another fresh MB solution to test the reusability/repeatability of the immobilized ZnO NW arrays. Figure 6a shows the degradation profiles of MB in the presence of the ZP films for four consecutive cycles. *C*/*C*_0_ on the vertical axis represents the ratio of the degraded concentration (*C*) to the initial concentration (*C*_0_) of the pollutants, which was calculated from the absorbance of the MB solution using the Bouguer–Lambert–Beer law [15, 40–42]. It is observed that the degraded percentages of MB after each of the four 3-h-long degradation experiments are 74.9%, 82.1%, 70.6%, and 71.4%, respectively. The reusability of this immobilized, floatable photocatalytic platform can be also evaluated by studying the degradation kinetics. According to a pseudo-first-order kinetic model with a dilute concentration, apparent reaction rate constant *k* follows − ln(*C*/*C*_0_) = *kt*, where *t* is the irradiation time [11, 12, 43, 44]. Therefore, *k* can be obtained from the slope of the linear plot of − ln(*C*/*C*_0_) versus *t* (see Fig. 6b). Figure 6c displays the corresponding degradation kinetics of the MB dye solution for the four consecutive cycles with *k* values of 0.008, 0.0091, 0.0071, and 0.007 min^−1^, respectively. These results confirm the reusability of the ZP film. In the immobilized photocatalytic system, the mechanical integrity of the photocatalytic nanostructure on the supporting substrates is important for reusability and long-term usage. Compared to other immobilized photocatalytic platforms that involve either deposition or direct growth of semiconducting nanomaterials onto the supporting substrate, the proposed approach allows a portion of the ZnO NWs to be embedded or anchored onto the PDMS film, which significantly promotes the mechanical robustness of the NWs and thus the reusability of the immobilized system. This reliable immobilization of the ZnO NW array was also observed by inspecting the remaining degraded solution. No NWs were found in the degraded solution after being centrifuged or filtered.

**Fig. 7:**
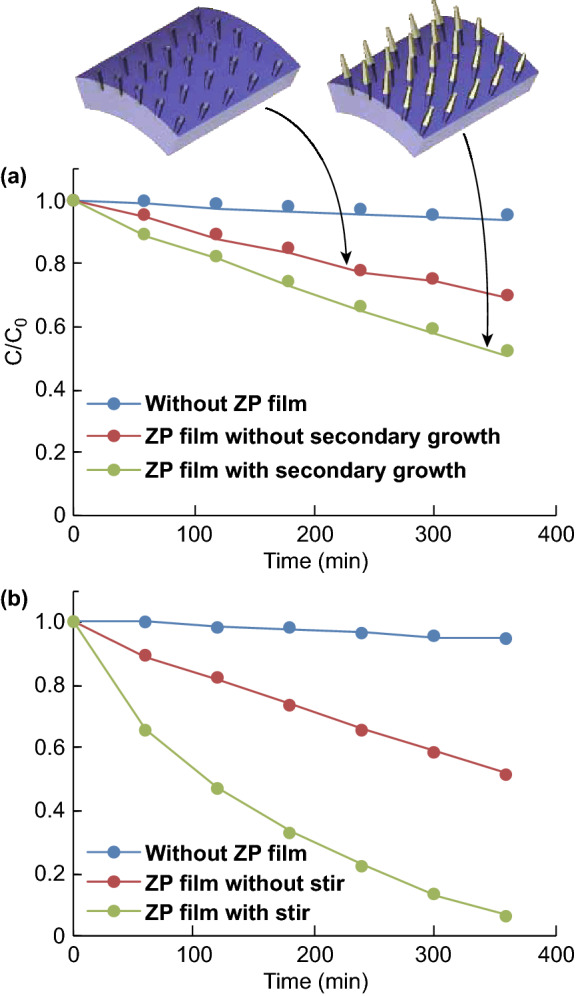
Degradation profiles of the MB dye (at 667 nm) **a** before/after the secondary growth and **b** with/without magnetic stirring

Many factors play key roles in photodegradation, including the reaction temperature, pH of the solution, active surface areas of catalysts, and mass transfer rates [45]. The last two factors are particularly important for an immobilized photocatalytic platform because catalysts fixed on the substrate present much smaller active surface areas compared to those in a slurry suspension-based catalytic system and the reactants need to diffuse to the substrate surface. Therefore, an immobilized photocatalytic platform typically suffers from low reaction rates and high mass transfer resistances. First, the effect of the catalyst surface area on the photodegradability can be evaluated by comparing two immobilized platforms with different wire lengths (schematically shown in Fig. 7a). It is anticipated that the ZP film with the secondary growth will have a larger surface area of the ZnO NWs exposed to the environment in comparison with that of the sample without the secondary growth and thus present a higher photocatalytic degradability. Figure 7a shows the photodegradation performance for these two immobilized platforms with and without the secondary growth of the ZnO NW array. As expected, the ZP film after the secondary growth exhibits a higher degradation rate (about 50% of MB degraded after 6 h) than the ZP film without the secondary growth (about 30% degraded after 6 h). The apparent reaction rate constant (*k*) is 0.0010 m^−1^ for the ZP film without the secondary growth and 0.0018 m^−1^ for one with the secondary growth. Because the degradation experiments were performed in an identical condition, the higher photodegradation performance of the ZP film with the secondary growth can be attributed to the larger active surface area from the longer length of the ZnO NWs—more light absorption and reaction sites for pollutant decomposition.

Secondarily, another important factor limiting the degradation performance of immobilized photocatalytic systems is a slow mass transfer rate, i.e., a sluggish transport of the reactants and products to and from the catalyst surface. The photocatalytically generated oxidants, such as hydroxyl radicals and super oxide ions, are short-lived species and available only in the vicinity of the immobilizing substrate. Thus, the overall chemical reactions are governed by the transport rate of the pollutants to (or byproducts from) the substrate. Without an external field, this transport is dependent merely on diffusion, which is sluggish. Here, we investigated the effect of mass transfer on the degradability of the ZP film with secondary ZnO NW growth by implementing magnetic stirring (at 700 rpm). As shown in Fig. 7b, the degradation rate is significantly improved when magnetic stirring is employed. Apparent reaction rate constant *k* increases from 0.0018 min^−1^ (without stirring) to 0.0074 min^−1^ (with stirring). It can be concluded from these two sets of experiments that the enhancement of the mass transfer rate has a more significant impact on the degradability of the ZP film. The combination of the ZP film with the secondary growth and active stirring is favored in the photodegradation experiments.

### Immobilized Platform for Piezocatalysis

**Fig. 8:**
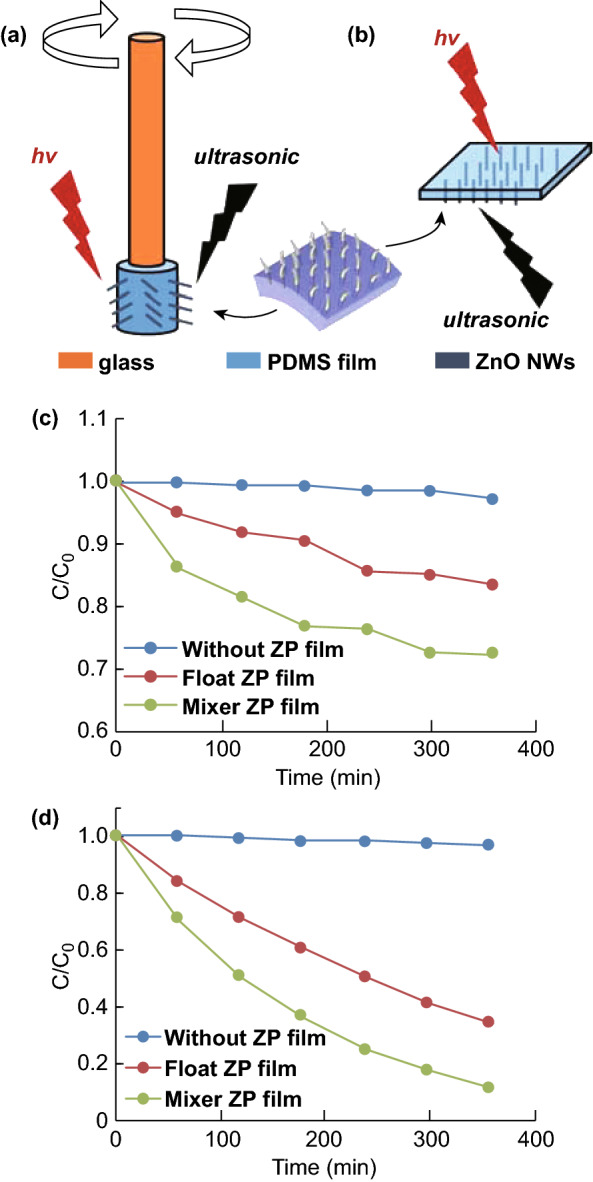
Schematics of **a** ZP film attached to a glass rod and **b** floating ZP film. Degradation profiles of the MB dye (at 667 nm) **c** without UV light irradiation and **d** with UV light irradiation

**Fig. 9:**
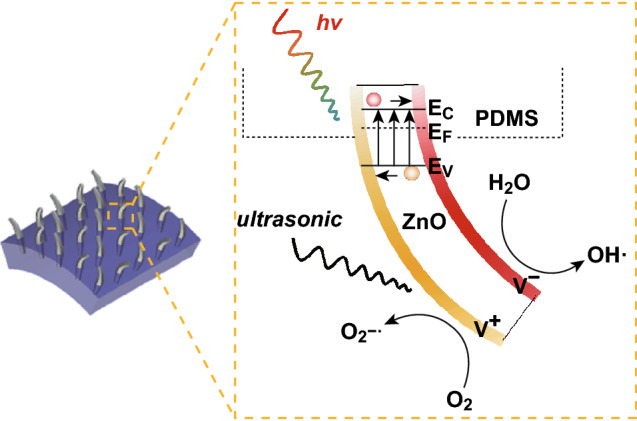
Schematics of the piezo-photocatalytic mechanism

The strong adhesion of the NWs to the substrate enables us to explore a novel application, i.e., an immobilized ZP film as a piezocatalyst. Degradation of organic pollutants by piezocatalysts has been previously demonstrated in a slurry (or suspension) form [46]. It is well known that ZnO is a piezoelectric material, i.e., electric charges are generated and accumulated in response to applied mechanical stresses. Because the ZnO NW array is securely adhered to the substrate, large stresses/strains can be applied to the ZP film, facilitating immobilized piezocatalysts. In detail, the ZnO is bent or stretched and mounted onto a circular rod, which is rotating with the rod and ultrasonicated at the same time. In comparison, another ZP film was floated atop the polluted water under only an applied ultrasonic field. Even more interestingly, the immobilized floating catalyst can be used to degrade pollutants rapidly. The schematics of the ZP films with/without ultrasonication/UV light irradiation for the investigation of piezocatalysis are illustrated in Fig. 8a, b, where the bended ZnO NWs are caused by ultrasonic-induced water pressure [47]. The piezocatalytic activity of the ZP film with vibration (no UV light irradiation) is shown in Fig. 8c. In the presence of an ultrasonic field, 15% of MB dye is decomposed by the floating ZP film (red line) in a dark condition, while the reference solution remains unchanged (blue line), proving the effectiveness of piezocatalysis to the MB dye. The degradability of this ZP film is increased significantly when it is further assisted by UV illumination (Fig. 8d, red line), confirming the piezo-photocatalytic mechanism of the ZnO NWs, as reported [48]. The piezo-photocatalytic mechanism and potential reactions are illustrated in Fig. 9. As can be seen, the piezocatalysis perform two functions in the degradation process, including excitation of the electron–hole pairs and separation of the photoinduced charge carriers. Owing to the existence of an increased number of charge carriers, more reactive oxidants are converted, hence elevating the capability of degradation eventually. It is known that the performance of a piezocatalyst is highly governed by the vibration frequency [48]. In comparison, a setup was built for a ZP film to further experience enhanced vibration frequency. In the experiment, a ZP film was wrapped around a glass rod, which was attached on a rotator, as presented in Fig. 8b. The fixed ZP film in the entire degradation process was ultrasonicated while being rotated (at 700 rpm) simultaneously. Consequently, the immobilized ZP film on the rod obtained a higher degradation rate than the floating one, which indicated that an increased vibration frequency was applied to the ZnO NWs. Likewise, the immobilized ZP film on the rod exhibited better degradability than the floating one under UV irradiation, which was mainly attributed to the enhancement of the piezo-photocatalytic efficiency. Other than the aforementioned factors, such as active surface areas of catalysts and mass transfer rate, the reaction temperature is usually considered as another primary aspect that can significantly affect the degradation rate. In piezocatalysis experiments, the employment of ultrasonication may induce a rapid heat-up localized on the ZnO NWs, further promoting the degradation process. So far, the complete decoupling of heat from the high-frequency vibrations for piezocatalysis is challenging for us. However, the system temperature was intended to maintain stability during the entire experiment. From this point of view, piezocatalysis as one dominant contribution in degradation is demonstrated.

### Photocatalysis of Crude Oil by Immobilized Platform

**Fig. 10:**
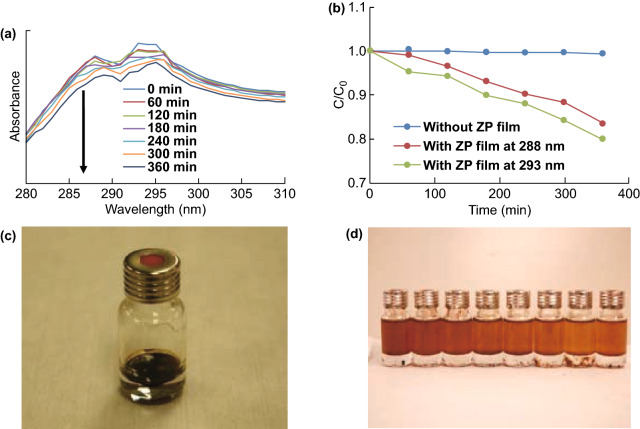
**a** UV–Vis spectrum of the crude oil. **b** Degradation profiles of the crude oil (at 288 and 293 nm). Photographs of the crude oil **c** before photodegradation and **d** after photodegradation

Until now, considerable efforts have been devoted to treat pollutants from industries and agriculture. In the field of photocatalysis, immobilized photocatalysts are regarded as promising candidates in removing floating pollutants (e.g., oil spills). In this study, crude oil was used as another probe pollutant to evaluate the photodegradation capability of the ZP films, where the decomposition was validated by monitoring the concentrations of particular components. Figure 10a shows the spectral changes of the crude oil measured using a UV–Vis spectrometer. As can be seen, the crude oil exhibits many characteristic peaks owing to its complex composition. All the peaks are weakened as the irradiation time increases, indicating that the various organic components are indeed degraded by the ZP film. To clearly analyze the degradation performance of the ZP films, the degradation profile of crude oil is plotted as function of the irradiation time for two peaks at 288 and 293 nm, respectively (Fig. 10b). The concentration of the crude oil is observed to decline around 20% in 6 h in the presence of the ZP film. In addition, the impact of UV light alone on decomposing this crude oil is found insignificant (Fig. 10b, blue line). The corresponding crude oil samples before and after the degradation are displayed in Fig. 10c, d. In Fig. 10c, a circular ZP film is placed on top of a crude oil layer, which has approached absorption/desorption equilibrium prior to the experiment. After a 6-h degradation process, a reduction in the oil concentration is clearly observed (see color gradient in Fig. 10d) after dilution by the same amount of petroleum ether, suggesting the valid degradation process by means of ZP films.

It is worth mentioning that a quantitative comparison of the ZP film and other photodegradation techniques (e.g., slurry system, immobilized photoreactors, and floatable photocatalysts) would be highly desirable. However, it is often not feasible to quantitatively compare these methods because the outcomes are highly dependent on the light source and intensity, ratio of the photocatalyst to the pollutant, and water volume and some other experimental conditions. Therefore, more future works will be done to make the comparison by employing the similar operation conditions as other techniques.

## Conclusion

In this study, we fabricated a new type of immobilized photocatalytic platform for photodegradation. This platform was proposed possessing a number of advantages: (1) avoided the contamination caused by photocatalyst re-suspension, (2) increased the utilization of the incident light, and (3) facilitated the mass transfer. According to the results, the floating ZP film exhibited a considerable degradability and reusability against MB dye. From the degradation mechanism, because the redox reaction only occurred at the interface of the solution and NWs, the longer exposed NW part resulted in a better photocatalytic performance. In addition, the ZP film, which moved energetically, also had a high degradability. In the experiment, we also investigated the piezocatalytic and photocatalytic properties of the ZP film. Meanwhile, a proper platform was proposed to effectively improve the piezocatalytic capability of the ZP film. Considering the practical challenge in treating floating pollutants, crude oil as a model pollutant was tested by the ZP film. The result confirmed that a slow but valid photocatalytic degradation process took place in the presence of the ZP film. For a better performance, a high-power light source needs to be introduced to accelerate the process. Sunlight irradiation and other types of pollutants need to be introduced to further evaluate the ZP film in future works.
